# Clinical features and outcome of Wilson's disease with generalized epilepsy in Chinese patients

**DOI:** 10.1111/cns.13373

**Published:** 2020-04-13

**Authors:** Rou‐Min Wang, Hao Yu, Guo‐Min Yang, Wan‐Qing Xu, Nan Xia, Yue Zhang, Wang Ni, Yi Dong, Zhi‐Ying Wu

**Affiliations:** ^1^ Department of Neurology and Research Center of Neurology in Second Affiliated Hospital, and Key Laboratory of Medical Neurobiology of Zhejiang Province Zhejiang University School of Medicine Hangzhou China; ^2^ Department of Neurology and Institute of Neurology Huashan Hospital Shanghai Medical College Fudan University Shanghai China

**Keywords:** Chinese, clinical features, generalized epilepsy, outcome, Wilson's disease

## Abstract

**Objective:**

Generalized epilepsy is rarely reported in patients with Wilson disease (WD) and lacks experience in clinical practice. We aim to provide better experience for the diagnosis and treatment for WD patients with epilepsy in the future.

**Methods:**

A retrospective study was performed in 13 Chinese WD patients with generalized epilepsy. Each patient was diagnosed with WD by clinical evaluation and genetic screening. Patients were given small doses of antiepileptic drugs (AEDs), followed by copper‐chelation therapy when the seizures stabilized. Clinical manifestations, brain imaging changes, and treatment and outcome after a long‐term follow‐up were analyzed.

**Results:**

Four out of 13 (30.8%) patients stopped taking copper‐chelation drugs for more than 1 year before they were admitted for epilepsy. The incidence of epilepsy of WD patients in our cohort is 1.43% (13/910), lower than those (4.5%‐5.9%) in other populations. After the attack of epilepsy, frontal lobes were the most common abnormalities (13/13, 100%) in patients, followed by brain stem (8/13, 61.5%) and thalamus (7/13, 53.8%). After a long‐term follow‐up, brain imaging and clinical manifestations of 8 (8/9, 88.9%) WD patients were significantly improved.

**Conclusions:**

We firstly described WD patients with generalized epilepsy in the Chinese population. WD patients with aggravation of neuropsychiatric symptoms are prone to occur epilepsy; thus, brain MRI should be performed regularly in those patients. Cortical abnormality in brain MRI is a warning sign of epilepsy. Irregular use of copper‐chelation drugs and excessive copper deposition in the brain may be the cause of seizures. Long‐term standardized treatment for WD can effectively prevent the extensive brain damage and reduce the incidence of epilepsy in WD patients.

## 1 INTRODUCTION

Wilson's disease (WD), one of the monogenic inherited diseases, is a kind of copper metabolic disturbance. The gene *ATP7B* mapping to chromosome13 (13q14.3) accounts for the disease, which encodes a P‐type ATPase of transporting copper.^1^ The deficiency of this protein prevents copper from being pumped out of the cell and therefore overloaded copper depositing in some specific organs, like liver, brain, cornea, and skeleton.^2^ In brain, copper is more likely to accumulate in the basal ganglia, especially near the region of lenticular nucleus, followed by the brain stem.^3^ The corresponding neuro‐phenotypes related to the functional areas will appear, such as parkinsonism, tremor, and dystonia. The clinical heterogeneity and complications of WD increase the difficulties in diagnosis and treatment.

Although WD has common clinical manifestations, epilepsy and other secondary symptoms should not be ignored in the progress of the disease. In WD, abnormal deposition of copper or copper‐induced damages could lead to brain function impairment and abnormal discharge of neurons, resulting in attacks of generalized epilepsy.^4^, ^5^ A rare event as it is, there is little experience in clinical practice. If the generalized epilepsy is not diagnosed and treated in time, the patients may progress into refractory epilepsy, missing the best opportunity for treatment, and their WD symptoms may deteriorate irreversibly.^6^

During the last 15 years, we had diagnosed 910 unrelated WD patients by screening *ATP7B* mutations and found that 13 of them had attacks of generalized epilepsy. Here, we reviewed their clinical manifestations, brain imaging changes, and treatment and outcome, aiming to provide better experience for the diagnosis and treatment of WD patients with epilepsy in the future.

## 2 MATERIALS AND METHODS

### 2.1 Subjects

All patients were recruited from the Second Affiliated Hospital of Zhejiang University School of Medicine and Huashan Hospital of Fudan University. The study was approved by the ethics committees of these two hospitals, and the informed consents were obtained from the patients or their guardians. Among these 13 patients, the mutations carried by eight patients (P1‐P8) and another 2 (P9‐P10) had been reported, respectively, in our two previous studies.^7^, ^8^ The remaining three patients (P11‐P13) were newly recruited between September 2017 and March 2019 from the Second Affiliated Hospital of Zhejiang University School of Medicine. The patient was assessed in detail using related laboratory tests, electroencephalography (EEG), magnetic resonance imaging (MRI), and abdominal ultrasound.

### 2.2 Mutation analysis

Extracting DNA from peripheral EDTA‐treated blood was performed using Blood Genomic Extraction Kit (Qiagen) according to the manufactures’ instructions. Sanger sequencing was performed on an ABI 3500xL Dx Genetic Analyzer (Applied Biosystems), and the procedure was described previously.^7^, ^8^ For patients who had only one variant detected by Sanger sequencing, we further performed multiplex ligation‐dependent probe amplification assay (MLPA) using the ATP7B MLPA kit (SALSA P098‐D1, MRC‐Holland).

## 3 RESULTS

### 3.1 Clinical characteristics of patients during the first visit

**FIGURE 1:**
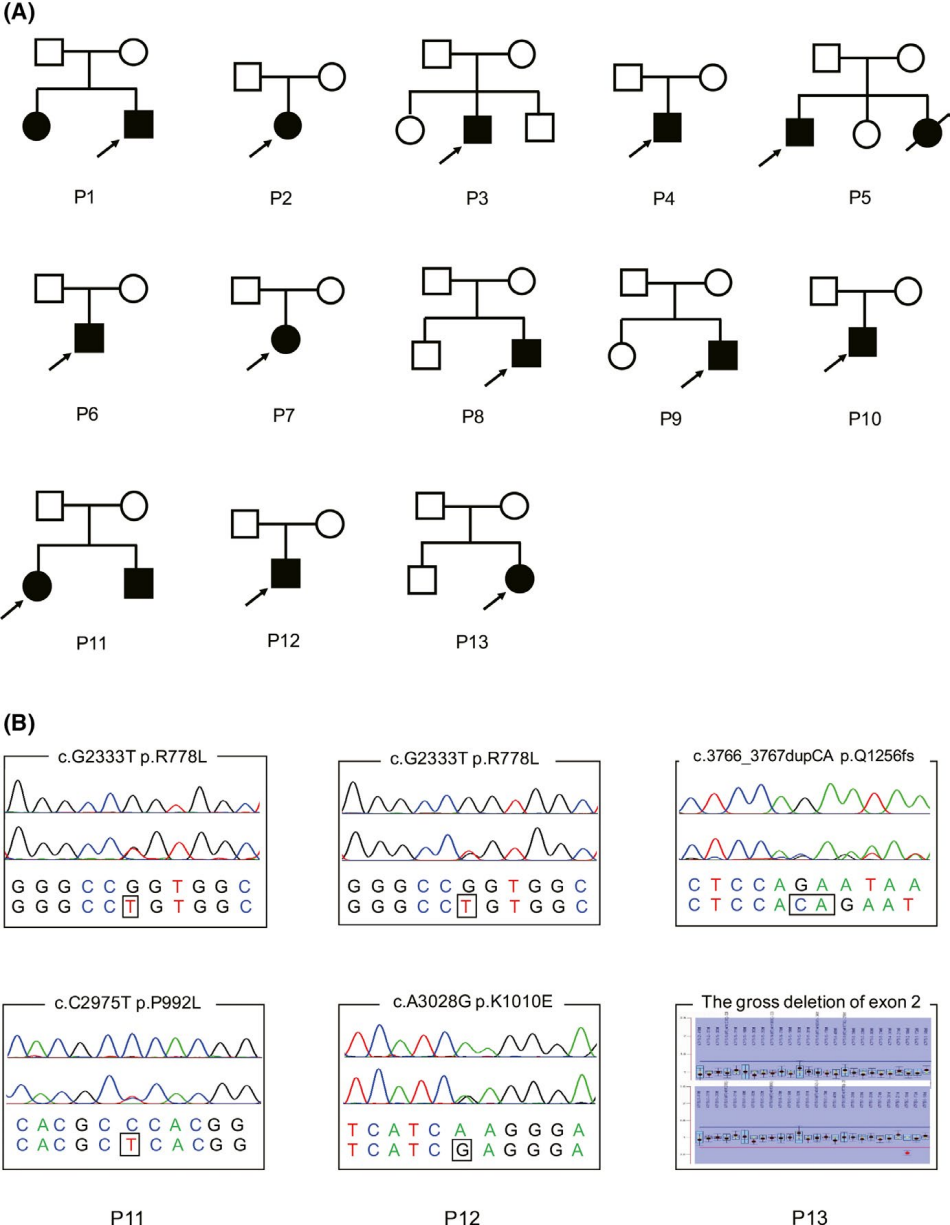
Pedigrees and mutation analysis. A, The pedigrees of 13 WD patients. Squares indicate males, circles indicate females, the black symbols indicate affected individuals, and arrows indicate the probands. B, Sequencing chromatograms and MLPA assay of three newly recruited patients (P11, P12, and P13). The upper panel depicts the reference sequence, and the lower panel represents the abnormal sequence

After analyzing 910 unrelated WD patients recruited between January 2004 and March 2019, we found that 13 (9 males and 4 females) of 910 (1.43%) patients were recorded to have generalized epilepsy. The pedigrees of them are shown in Figure 1A, and genetic analysis of 3 newly recruited patients (P11‐P13) is shown in Figure 1B. The genetic variation and the clinical characteristics of these 13 patients during the first visit are shown in Table 1. The mean age at onset (AAO) of them was 13.4 years (range 7‐21 years). The Kayser‐Fleischer (K‐F) rings and low levels of serum ceruloplasmin were observed in all patients. The index of urinary 24‐hour copper excretion before the copper‐chelation therapy ranged from 95 to 827 µg/24 h (reference: 0‐60 µg/24 h). Initial symptoms were neurological abnormalities in 10 patients, hepatic abnormalities in 2 patients, and osseomuscular abnormalities in 1 patient. Over time, patients progressed into typical and mixed manifestations of WD, and all 13 patients presented neurological abnormalities, including various degrees of dysarthria, dystonia, and parkinsonism. In addition, 11 patients had different levels of mental disorders, 5 patients had gastrointestinal symptoms, and 3 patients complained of arthralgia.

**TABLE 1:** Clinical characteristics of 13 patients during the first visit

Case	Gender	*ATP7B* variants	AAO (y)	Initial symptoms	K‐F rings	CP	24‐hour urine copper^a^	Neurological symptoms	Digestive symptoms	Other symptoms
P1	M	p.N1270S c.2575 + 2T＞A	14	N	+	<20	377	DS, TR, PA	−	Irritability
P2	F	p.P992L p.P992L	8	H	+	40	N/A	DS, PA, DO, DP, DR	−	Dysphoria, irritability
P3	M	p.E332 p.Q1372	7	H	+	30	446	DS, DO, AT, DR	Limb weakness jaundice, abdominal distension	Arthralgia
P4	M	p.R778L p.P992L	21	N	+	<20	363	DS, TR, PA, DO	−	Euphoria, paranoia, irritability
P5	M	p.P992L p.I930del	19	O	+	<20	270	DS, TR, PA, DO	−	Arthralgia
P6	M	p.I390V + p.A874V c.1708‐1 G＞C	17	N	+	N/A	N/A	DS, DO, AT, DP, DR	Rash, jaundice	Irritability
P7	F	p.R778L p.L1088S	17	N	+	<20	N/A	DS, TR, PA	−	Irritability, suspicion
P8	M	p.R778L p.T935M	13	N	+	25	281	DS, DO, PA	−	Stubbornness, unsociability
P9	M	p.R778L p.V1216M	14	N	+	<20	N/A	DS, PA, DR	−	Depression, unsociability
P10	M	p.R778L p.S738H	20	N	+	42	827	DS, DO, TR, DP	Jaundice	Euphoria, irritability
P11	F	p.R778L p.P992L	12	N	+	23	N/A	DS, TR, PA	Limb weakness	Irritability
P12	M	p.R778L p.K1010T	18	N	+	<20	95	TR, DO	−	Arthralgia, irritability
P13	F	p.Q1256fs Deletion of Exon2	11	N	+	<20	222	DS, TR, DO, PA, DR, AT	Limb weakness	Cognitive decline

Abbreviations: −, negative; +, positive; AAO(y), age at onset (years old); AT, ataxia; CP, ceruloplasmin(mg/L); DO, dystonia; DP, dysphagia; DR, drooling; DS, dysarthria; F, female; H, hepatic symptoms; K‐F rings, Kaiser‐Fleischer's rings; M, male; N, neurologicalsymptoms; N/A, not available; O, osseomuscular symptoms; PA, parkinsonism; TR, tremor.

^a^ The index of 24‐hour urine copper (µg/24 h) was collected before each patient's copper‐chelation therapy.

### 3.2 Clinical features of patients during hospitalization for generalized epilepsy

When generalized epilepsy occurred, the patient was admitted to the hospital. None of the patients had ascites. The clinical characteristics of these 13 patients during hospitalization for epilepsy are shown in Table 2. Assessment of liver function including Child‐Pugh classification is shown in Table 3. Before epilepsy occurred, seven patients had a presentiment. Two patients (P2 and P11) presented spontaneous laughter or crying with no reason, and two (P7 and P10) complained of hallucinations. In addition, P3, P6, and P8 had formication, personality change, rant, and rave, respectively. During the first attack of epilepsy, all patients began with generalized tonic‐clonic seizure (GTCS), except P3 with tonic seizure. The frequency of seizures is quite different and varies from one to several times per day. There is no regularity or similarity in the course of the disease or the involved parts. It is worth mentioning that five patients (P2, P5, P6, P7, and P11) had various kinds of transient accompanying symptoms like rash or jaundice during their episodes.

**TABLE 2:** Clinical characteristics of 13 patients during hospitalization for generalized epilepsy

Case	Age	Presentiment	Frequency	Duration (min)	Involved parts	Other symptoms	Involvement on brain MRI
P1	17	−	Several times/d	10	R	−	BG, BS, TH, FL
P2	15	Spontaneous laughter or crying	Several times/d	2	L	Jaundice	BG, BS, TH, FL
P3	20	Formication	Six times/y	N/A	N/A	N/A	BG, FL, PL
P4	24	−	Four times/half year	3‐5	B	−	BG. FL, PL
P5	52	−	Seven times/mo	2	B	Dizziness	BG. FL, PL
P6	20	Personality change	Two times/mo	N/A	B	Rash	BG, FL, BS, TH
P7	19	Hallucination	Several times/d	2	N/A	Rash	BG, FL, TL
P8	33	Rant and rave	Two times/d	20	B	−	BG, FL BS
P9	21	−	Two times/mo	N/A	L	−	BG, TH, BS, FL, TL
P10	23	Hallucination	Three times/d	1	R	−	BG, TH, BS, FL, TL
P11	27	Spontaneous laughter or crying	Only once	20	B	Rash	BG, FL
P12	25	−	Several times/d	2	B	−	BG, BS, TH, FL
P13	14	−	Five times/d	8	B	−	BG, FL, TH, CB, BS

Abbreviations: −, negative; B, the whole body; BG, basal ganglia; BS, brain stem; CB, cerebellum; FL, front lobe; H, hepatic subscale; L, left body; N, neurological subscale; N/A, not available; P, psychiatric subscale; PL, parietal lobe; R, right body; TH, thalamus; TL, temporal lobe.

**TABLE 3:** Child‐Pugh classification and abdominal ultrasonography of 13 patients

Case	Stage of hepatic encephalopathy	Serum bilirubin (µmol/L)	Serum albumin (g/L)	Prothrombin time(s)	Totally score	Class	Abdominal ultrasonography
P1	None	12.1	69	13.8	5	A	Hepatic diffuse disease
P2	I	7.8	14.3	16.2	9	B	Hepatic diffuse disease
P3	None	16.1	51	14	5	A	Hepatic diffuse disease, splenomegaly
P4	None	12	47	11.4	5	A	Hepatic diffuse disease
P5	None	12	45	12.2	5	A	Hepatic diffuse disease, splenomegaly
P6	I	19	42	16.2	7	B	Hepatic diffuse disease
P7	None	17.8	47	11.6	5	A	Liver cirrhosis
P8	None	12.2	44	12.9	5	A	Splenomegaly
P9	None	11.5	44	15	6	A	Hepatic diffuse disease, splenomegaly
P10	I	17.6	36.1	15.4	7	B	Hepatic diffuse disease
P11	None	12	40.7	12.1	5	A	Liver cirrhosis
P12	None	16.9	40.7	13.9	5	A	Normal
P13	None	9.5	38.9	13.6	5	A	Hepatic diffuse disease, splenomegaly

Note

Grade A = 5‐6 points; grade B = 7‐9 points; grade C = ≥10 points.

**FIGURE 2:**
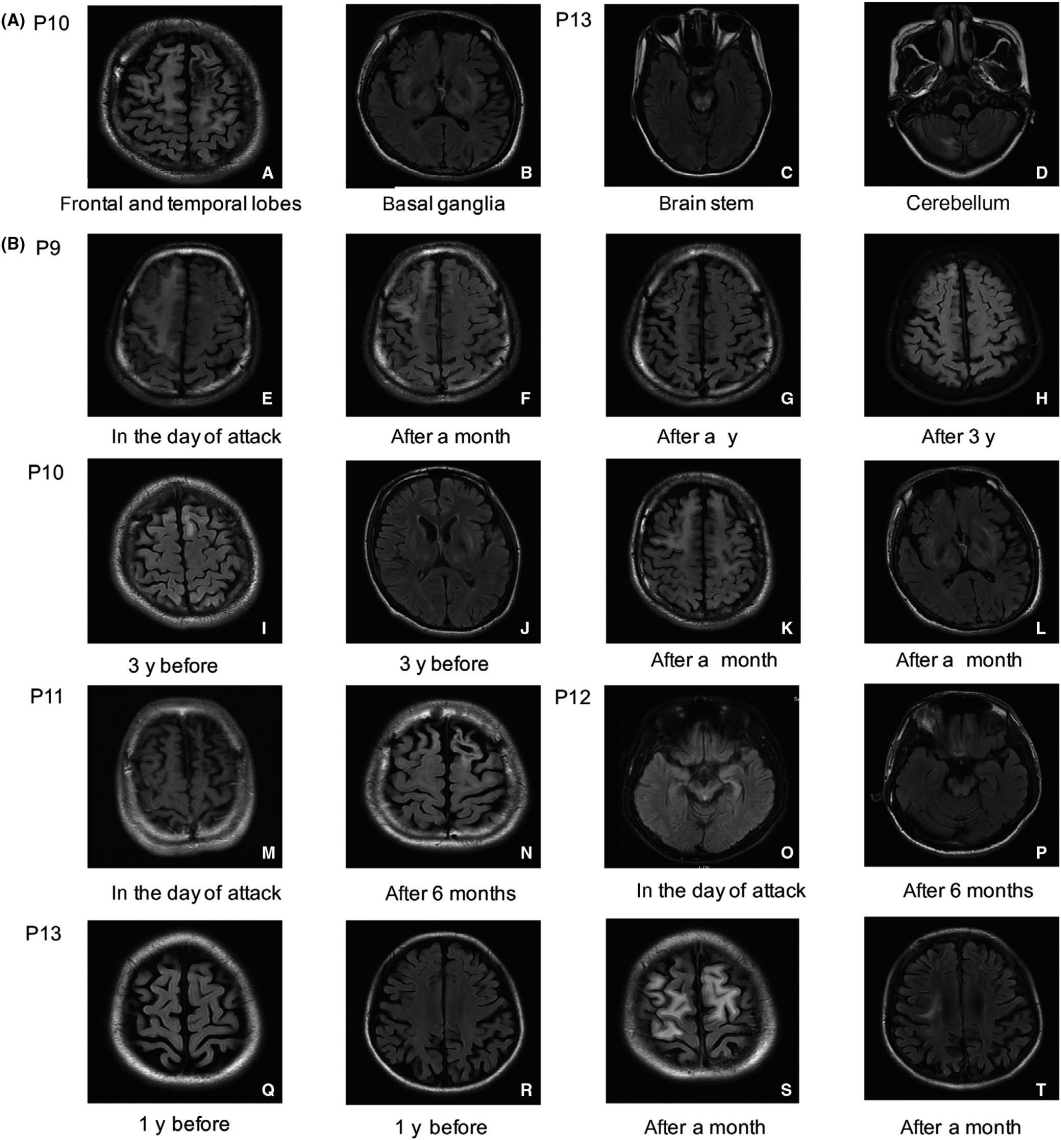
Brain MRIs of WD patients suffered from generalized epilepsy. A, Typical involvements of two patients (P10 and P13) including T2‐weighted FLAIR brain MRIs during the hospitalization for generalized epilepsy. B, Brain MRIs of five WD patients (P9‐13) before occurring epilepsy and after treatment. The figures (e‐h) were the 3‐y follow‐ups of P9 focused on the same level in the cortex. The figures (i, k) and (j, l) were the contrast of basal ganglia and cortex of P10 before and after seizures. The P11 and P12 had obvious imaging changes in half a year according to the figures (m, n) and figures (o, p). The last two figures (s, t) were the new lesions in cortex of P13 during 1 y

Seven patients (P1, P4, P5, P7, P11, P12, and P13) underwent the EEG testing. The regular EEG captured θ wave on single or bilateral sides and extensive δ wave in 4 patients (P4, P5, P7, and P13), and normal rhythm and frequency in the other 3 patients (P1, P11 and P12). The brain MRI abnormalities of 13 patients after attacking of epilepsy are summarized in Table 2. In addition to the basal ganglia, frontal lobes were the most common abnormalities (13/13, 100%), followed by brainstem (8/13, 61.5%) and thalamus (7/13, 53.8%). In the cortex, the rate of abnormalities in the parietal or temporal lobes was similar (3/13, 23.1%). In addition, P13, whose brain MRI showed extensive white matters, had some additional high signals in the cerebellum (Figure 2A).

As a typical example, when he was 20 years old, P10 was initially diagnosed with WD due to difficulty in writing and speaking, and received copper‐chelation treatment. He had status epilepticus at the age of 23 and was admitted to our center. According to his mother, he had been off his medication for about 7 months. He had hallucinations before the seizure. The frequency of seizures was about three times per day. In acute episode, the patient experienced intense contraction in his right limbs with eyes gazing to the left, following the alternating myoclonus, which lasted for nearly 1 minute. Then, the patient remained unconscious for nearly 20 minutes before fully waking up and suffered a long time of flaccid paralysis. We examined the patient and found that muscle strength was grade 0 in the right limbs and grade 4 in the left limbs, and the tendon reflexes of the extremities were reduced. During his hospitalization, several attacks of tonic seizures were also captured.

### 3.3 Treatment and outcome

Once the diagnosis of WD is confirmed, copper‐chelation treatment begins. Copper‐chelation drugs used by patients before epilepsy are summarized in Table 4. Three patients (P2, P4, and P6) were treated with large doses of D‐penicillamine (DPA) at their first visits. Four patients (P7, P9, P10, and P13) stopped taking copper‐chelation drugs for more than 1 year before they were admitted to hospital for epilepsy. The rest of the patients followed the doctor's advice and had no adverse reactions. During the hospitalization for generalized epilepsy, copper‐chelation drugs were suspended for a while and antiepileptic drugs (AEDs) were prescribed to ensure the effective control of seizures in a short time. As shown in Table 4, diazepam (DZP) was used to control status epilepticus and other AEDs like levetiracetam (LEV), carbamazepine (CBZ), and valproate (VPA) were applied to prevent the patients' seizures from recurring. No serious side effect was found in these patients. For example, AEDs (LEV 250 mg b.i.d. and VPA 500 mg q.d.) were given to P12 for months because of his multiple MRI abnormalities even though he had only one attack. When his condition was stable and no more seizures occurred within 1 month, the copper‐chelation drugs were added to ensure the process of WD treatment.

**TABLE 4:** Treatment and outcome of 13 patients

Case	Medication before seizures	Antiepileptic drugs	Symptoms of Wilson's disease (follow‐up)
P1	DPA 62.5 mg Tid	LEV 250 mg Bid	Tremor disappeared with improved speech (12 mo after treatment)
P2	DPA 375 mg Tid	LEV 250 mg Bid	Dead (1 mo after treatment)
P3	DPA 250 mg Tid	TPM 500 mg Bid	Lost
P4	DPA 375 mg Tid	VPA 500 mg Qd	Motor disorders improved but emotion problem existed (36 mo after treatment)
P5	DPA 250 mg Tid	CBZ 200 mg Bid	Speech function improved and joint pain disappeared (24 mo after treatment)
P6	DPA 375 mg Tid	N/A	Dead (6 mo after treatment)
P7	Medication Free	TPM 500 mg Bid	Lost
P8	DMSA 500 mg Tid	N/A	Motor disorders improved with better mood (12 mo after treatment)
P9	Medication free	LEV 250 mg Bid	Tremor disappeared with improved speech (12 mo after treatment)
P10	Medication free	LEV 250 mg Bid	No obvious improvement (10 mo after treatment)
P11	DMSA 500 mg Bid	LEV 250 mg Bid	Mental symptoms disappeared with improved speech(6 mo after treatment)
P12	DMSA 500 mg Bid	LEV 250 mg Bid VPA 500 mg Qd	Tremor disappeared (9 mo after treatment)
P13	Medication free	CBZ 300 mg Bid	Motor disorders improved but blurred speech persisted (8 mo after treatment)

Abbreviations: Bid, twice a day; CBZ, Carbamazepine; DMSA, Dimercaptosuccinic acid; DPA, D‐penicillamine; LEV, Levetiracetam; N/A, not available; Qd, once a day; Tid, three times a day; TPM, Topiramate; VPA, Valproate.

In the long‐term follow‐up, two patients (P2, P6) died of irreversible neurological manifestations and poor physical conditions, two (P3, P7) were lost for some reasons, and the rest (P1, P4, P5, P8 to P13) were followed up at our center. There was no recurrence in 5 patients (P8, P9, P5, P11, and P12), but 4 patients (P1, P4, P10, and P13) had relapse. P1 manifested complex partial epilepsy sometimes. P4 had a recurrence of generalized epilepsy after 2 years. P10 had epileptic seizures and myoclonic epilepsy from time to time and gradually developed into refractory epilepsy. P13 had some attacks of simple partial seizures in her left upper extremity, lasting 30 seconds each time. Six patients (6/7, 85.7%) had not suffered from generalized epilepsy after treatment and had different degrees of improvement in neuropsychiatric symptoms after a series of strict treatment (Table 4). The clinical symptoms of P10 (1/9, 11.1%) did not improve due to the refusal of the drug, and the patient ended up bedridden.

Brain MRIs of patients before epilepsy and after treatment are shown in Figure 2B. Unified Wilson's Disease Rating Scale (UWDRS), as an effective tool to monitor disease progression, was applied in patients’ hospitalization for seizures.^9^ After 6 months of double WD treatment, except the aggravation of neurological manifestations (125‐142) and psychiatric symptoms (4‐23) of P10, the other four cases (P9, P11, P12, and P13) had varying degree improvement in both neuropsychiatric symptoms and hepatic symptoms. As a positive example, P9 was improved greatly by 3‐year continuous treatment. With long‐term small dose of LEV, the patient had no recurrence of seizures. The lesion area of cortex was remarkably reduced after 6‐month treatment and returned to normal 1 year later. Moreover, it was almost invisible at the same area after 3‐year treatment.

## 4 DISCUSSION

Epilepsy occurred in WD patients are rare with only a few reports of secondary seizures in WD patients.^10^ In the current study, we firstly reported Chinese WD patients with generalized epilepsy and described in detail the clinical data of 13 unrelated patients. Compared to the generalized epilepsy rate (4.5%‐5.9%) of WD patients previously reported, ^6^, ^11^, ^12^ the incidence of epilepsy (1.43%, 13/910) in our WD patients is lower. Our patients had multiple damages of organs, including brain, liver, and joint, and showed complex manifestations. The index of urinary 24‐hour copper excretion at the time of initial diagnosis was at a high level, indicating a certain deposition of copper in the body. It is worth mentioning that 11 of 13 patients (84.6%) had psychiatric symptoms, such as irritability and unsociability, indicating extensive multiple injuries in the brain and prone to epilepsy. More attention and long‐term follow‐up should be given to these patients with severe neuropsychiatric phenotypes.

In this study, single AED or combination of two AEDs could control the seizures of most patients (12/13). The transition from two cases (P1 and P13) to partial epilepsy indicates that part of the damage is persistent, and AEDs should be used in a long term with small dose. Because patients had extensive brain damage, we do not recommend stopping AEDs in a short time. The evidence of drug withdrawal should be strictly in accordance with the guide.^13^ In addition, the traditional liver enzyme‐inducing AEDs like VPA should be used as little as possible and newer AEDs such as LEV are safer to WD patients with epilepsy.^14^ Continuous copper‐chelation therapy should be added on the basis of seizures control in clinical treatment. In the long‐term follow‐up, the clinical manifestations and drug reactions should be monitored continuously.

Positive EEG results can help doctors identify seizures attack and find the epileptic foci.^15^ In our patients, only 4 of 7 patients with EEG had positive results of extensive slow wave damage, which may be attributable to the low sensitivity and timeliness of regular EEG.^16^ Besides, hidden lesions and fewer episodes could result in negative results. Therefore, video EEG and multiple tests should be preferred in WD patients with epilepsy.

T2‐weighed FLAIR MRI is a preferred choice to find the associated lesions.^17^ Abnormal copper deposition in lenticular nucleus is the most common in WD patients.^18^ In a previous MRI study of WD, thalamus, midbrain, and pons are also regarded as common damaged areas.^19^ In our patients, in addition to basal ganglia, the frontal lobe in cortical was also the most common damaged area (100%), followed by brain stem (61.5%), thalamus (53.8%), and parietal and temporal lobes (23.1%). Cerebellar injury is believed to occur in 4.4%‐5.9% of WD patients^3^, ^20^ and occurred in one of our patients (7.7%). By comparison of brain MRI between WD patients before seizures, after seizures, and after treatment, the abnormalities caused by other diseases were excluded and some hidden lesions were found, which are helpful for the diagnosis. The increase or decrease of the signals in MRIs can reflect the course of the disease. Therefore, brain MRI can help to evaluate the progress of seizures and adjust the treatment in WD patients.

Although the brain MRIs have shown cortical and thalamic damages in patients with seizures, the origin of the seizures is difficult to determine and the mechanism underlying the extensive brain damage is not clear. There are many conjectures about the destruction of copper homeostasis in the body, such as copper overload, drug abuse, or quick discharge of copper. Abnormal neuron discharges in the cortex may be caused by copper changes or copper‐mediated damages such as over oxidative stress, proinflammatory cytokines, and glutamate activation.^6^ The cerebello‐thalamo‐cortical network has been proved to be able to explain some manifestations of Parkinson's disease and may be related to the cortical cell damage to a certain extent.^21^ Excessive intake of copper in the early stage or discharge of too much copper at one time may lead to copper transfer into other areas.^22^ In addition, copper‐chelation drugs may be another main cause of the seizures in WD patients. It is reported that DPA may lead to elevated free copper concentration and enhanced oxidative stress in some parts of the body, resulting in irreversible neurological impairment.^23^ Some first‐generation antipsychotic drugs like chlorpromazine can also increase the risk of seizures, due to the neurotransmitter and neuropeptide alterations.^24^, ^25^

## 5 CONCLUSIONS

In summary, we firstly described 13 WD patients with generalized epilepsy in the Chinese population. The incidence of epilepsy of WD patients (1.43%) is lower than those (4.5%‐5.9%) of previous reports about other populations. Excessive copper deposition in the brain and irregular use of copper‐chelation drugs may be the cause of epilepsy attacks. Long‐term standardized treatment for WD can effectively prevent the extensive brain damage and reduce the incidence of epilepsy in WD patients.

## CONFLICT OF INTEREST

The authors declare no conflict of interest.

## ETHICAL APPROVAL

We confirm that we have read the Journal's position on issues involved in ethical publication and affirm that this report is consistent with those guidelines.
